# 2-Amino-4,6-dimethyl­pyrimidin-1-ium chloride

**DOI:** 10.1107/S1600536812046569

**Published:** 2012-11-17

**Authors:** Hui-Ling Hu, Chun-Wei Yeh

**Affiliations:** a Department of Hospitality Management, Taoyuan Innovation Institute of Technology, Jhongli 32091, Taiwan; b Department of Chemistry, Chung-Yuan Christian University, Jhongli 32023, Taiwan

## Abstract

In the title compound, C_6_H_10_N_3_^+^·Cl^−^, the cation is essentially planar with an r.m.s. deviations of the fitted atoms of 0.008 Å. In the crystal, adjacent ions are linked by weak N—H⋯Cl hydrogen bonds involving the pyrimidine and amine N atoms, forming a three-dimensional network. C—H⋯π inter­actions between the methyl and pyrimidine groups and π–π stacking [centroid–centroid distance = 3.474 (1) Å] between parallel pyrimidine ring systems are also observed.

## Related literature

For the crystal structures of 2-amino­pyrimidinium salts with other anions, see: Cheng *et al.* (2010) ▶; Eshtiagh-Hosseini *et al.* (2010 ▶); Hu & Yeh (2012 ▶).
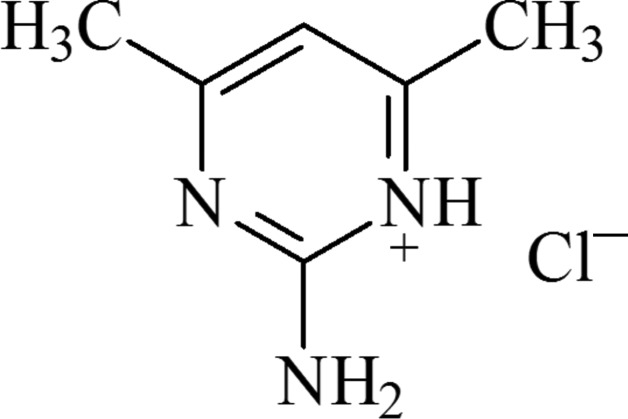


## Experimental

### 

#### Crystal data

C_6_H_10_N_3_^+^·Cl^−^
*M* *_r_* = 159.62
Monoclinic, 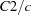

*a* = 16.372 (4) Å
*b* = 8.795 (2) Å
*c* = 12.007 (3) Å
β = 108.133 (5)°
*V* = 1642.9 (8) Å^3^
*Z* = 8
Mo *K*α radiation
μ = 0.40 mm^−1^
*T* = 273 K
0.4 × 0.4 × 0.3 mm

#### Data collection

Bruker APEXII CCD area-detector diffractometer
Absorption correction: multi-scan (*SADABS*; Bruker, 2000 ▶) *T*_min_ = 0.869, *T*_max_ = 0.982
5044 measured reflections
1620 independent reflections
1008 reflections with *I* > 2σ(*I*)
*R*_int_ = 0.046

#### Refinement

*R*[*F* ^2^ > 2σ(*F* ^2^)] = 0.039
*wR*(*F* ^2^) = 0.105
*S* = 0.90
1620 reflections
93 parameters
H-atom parameters constrained
Δρ_max_ = 0.18 e Å^−3^
Δρ_min_ = −0.17 e Å^−3^

### 

Data collection: *APEX2* (Bruker, 2010 ▶); cell refinement: *SAINT* (Bruker, 2010 ▶); data reduction: *SAINT*; program(s) used to solve structure: *SHELXS97* (Sheldrick, 2008) ▶; program(s) used to refine structure: *SHELXL97* (Sheldrick, 2008) ▶; molecular graphics: *DIAMOND* (Brandenburg, 2010 ▶); software used to prepare material for publication: *SHELXL97*.

## Supplementary Material

Crystal structure: contains datablock(s) I, global. DOI: 10.1107/S1600536812046569/gw2128sup1.cif

Structure factors: contains datablock(s) I. DOI: 10.1107/S1600536812046569/gw2128Isup2.hkl

Supplementary material file. DOI: 10.1107/S1600536812046569/gw2128Isup3.cml

Additional supplementary materials:  crystallographic information; 3D view; checkCIF report

## Figures and Tables

**Table 1:** Hydrogen-bond geometry (Å, °) *Cg1* is the centroid of the C1–C4/N2/N3 ring.

*D*—H⋯*A*	*D*—H	H⋯*A*	*D*⋯*A*	*D*—H⋯*A*
N1—H1*A*⋯Cl^i^	0.86	2.42	3.260 (2)	167
N1—H1*B*⋯Cl^ii^	0.86	2.57	3.262 (2)	138
N2—H2*N*⋯Cl	0.86	2.22	3.042 (2)	161
C5—H5*A*⋯*Cg*1^iii^	0.96	3.00	3.446 (3)	110

Symmetry codes: (i) 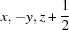; (ii) 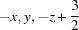; (iii) 
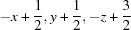
.

**Fig. 1.**
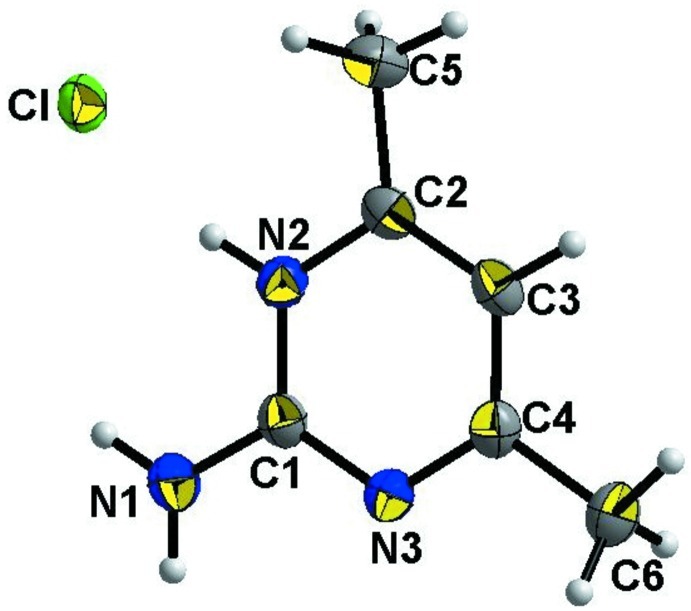
An ORTEP view of the title compound with the atom-labelling scheme.Thermal ellipsoids are drawn at 30% probability level, and H atoms are represented by small spheres of arbitrary radii.

**Fig. 2.**
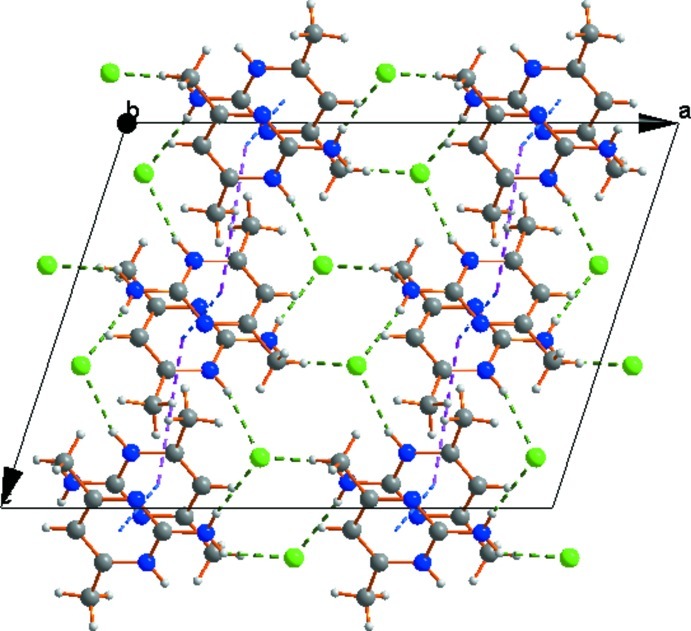
The packing diagram shows the N—H···Cl and C—H···pi hydrogen bonds and pi—pi stacking interactions forming the three-dimensional net.

## supplementary crystallographic information

### Comment

There are several supramolecular structures containing 2-aminopyrimidinium cations with other anions constructed by hydrogen bonds (Cheng, *et al.* 2010; Eshtiagh-Hosseini, *et al.* 2010; Hu, *et al.* 2012). The asymmetric unit of the title molecule, C_6_H_10_N_3_^+^, Cl^-^, consists a mono-protonated 2-amino-4,6-dimethylpyrimidine and one chloride anion (Fig. 1). The protonated pyrimidine groups are flat and these carbon/nitrogen atoms of mean devition from plane are 0.008 Å. The cations and anions are interlinked through N—H···Cl hydrogen bonds which are found between the H atoms bound to the pyrimidine and amine N atoms and the chloride anions showing the three-dimensional net (Fig. 2, Tab. 1). In the crystal, the weak C—H···pi interactions between the methyl and pyrimidinyl groups and the pi···pi stacking between parallel pyrimidine ring systems are observed, respectively [3.474 (1) Å], while *Cg1* is the centers of C1—C4/N2—N3.

### Experimental

An aqueous solution (5.0 ml) of zinc chloride (1.0 mmol) was layered carefully over a methanolic solution (5.0 ml) of 2-amino-4,6-dimethylpyrimidine (2.0 mmol) in a tube. Yellow crystals were obtained after several weeks. These were washed with methanol and collected in 83.5% yield.

### Refinement

H atoms bound to C atoms were placed in idealized positions and constrained to ride on their parent atoms, with C—H = 0.93 - 0.96 Å and N—H = 0.86 Å, and with *U*_iso_(H) = 1.2 or 1.5 *U*_eq_(C/N).

### Crystal data

**Table:**

C_6_H_10_N_3_^+^·Cl^−^	*F*(000) = 672
*M**_r_* = 159.62	*D*_x_ = 1.291 Mg m^−^^3^
Monoclinic, *C*2/*c*	Mo *K*α radiation, λ = 0.71073 Å
Hall symbol: -C 2yc	Cell parameters from 1118 reflections
*a* = 16.372 (4) Å	θ = 2.7–22.9°
*b* = 8.795 (2) Å	µ = 0.40 mm^−^^1^
*c* = 12.007 (3) Å	*T* = 273 K
β = 108.133 (5)°	Block, yellow
*V* = 1642.9 (8) Å^3^	0.4 × 0.4 × 0.3 mm
*Z* = 8	

### Data collection

**Table:**

Bruker APEXII CCD area-detector diffractometer	1620 independent reflections
Radiation source: fine-focus sealed tube	1008 reflections with *I* > 2σ(*I*)
Graphite monochromator	*R*_int_ = 0.046
phi and ω scans	θ_max_ = 26.0°, θ_min_ = 2.6°
Absorption correction: multi-scan (*SADABS*; Bruker, 2000)	*h* = −19→20
*T*_min_ = 0.869, *T*_max_ = 0.982	*k* = −10→10
5044 measured reflections	*l* = −14→11

### Refinement

**Table:**

Refinement on *F*^2^	Primary atom site location: structure-invariant direct methods
Least-squares matrix: full	Secondary atom site location: difference Fourier map
*R*[*F*^2^ > 2σ(*F*^2^)] = 0.039	Hydrogen site location: inferred from neighbouring sites
*wR*(*F*^2^) = 0.105	H-atom parameters constrained
*S* = 0.90	*w* = 1/[σ^2^(*F*_o_^2^) + (0.0545*P*)^2^] where *P* = (*F*_o_^2^ + 2*F*_c_^2^)/3
1620 reflections	(Δ/σ)_max_ < 0.001
93 parameters	Δρ_max_ = 0.18 e Å^−^^3^
0 restraints	Δρ_min_ = −0.17 e Å^−^^3^

### Special details

**Table:**

Geometry. All e.s.d.'s (except the e.s.d. in the dihedral angle between two l.s. planes) are estimated using the full covariance matrix. The cell e.s.d.'s are taken into account individually in the estimation of e.s.d.'s in distances, angles and torsion angles; correlations between e.s.d.'s in cell parameters are only used when they are defined by crystal symmetry. An approximate (isotropic) treatment of cell e.s.d.'s is used for estimating e.s.d.'s involving l.s. planes.
Refinement. Refinement of *F*^2^ against ALL reflections. The weighted *R*-factor *wR* and goodness of fit *S* are based on *F*^2^, conventional *R*-factors *R* are based on *F*, with *F* set to zero for negative *F*^2^. The threshold expression of *F*^2^ > σ(*F*^2^) is used only for calculating *R*-factors(gt) *etc*. and is not relevant to the choice of reflections for refinement. *R*-factors based on *F*^2^ are statistically about twice as large as those based on *F*, and *R*- factors based on ALL data will be even larger.

### Fractional atomic coordinates and isotropic or equivalent isotropic displacement parameters (Å^2^)

**Table:**

	*x*	*y*	*z*	*U*_iso_*/*U*_eq_	
Cl	0.06044 (4)	0.20186 (8)	0.63062 (5)	0.0635 (3)	
C1	0.19281 (13)	0.0360 (3)	0.9384 (2)	0.0456 (6)	
C2	0.28190 (14)	0.1835 (2)	0.8578 (2)	0.0476 (6)	
C3	0.35098 (14)	0.1274 (3)	0.9429 (2)	0.0524 (6)	
H3A	0.4063	0.1584	0.9477	0.063*	
C4	0.33775 (13)	0.0227 (3)	1.0228 (2)	0.0490 (6)	
C5	0.28542 (16)	0.2952 (3)	0.7666 (2)	0.0639 (7)	
H5A	0.2522	0.3834	0.7718	0.096*	
H5B	0.2623	0.2499	0.6905	0.096*	
H5C	0.3440	0.3244	0.7785	0.096*	
C6	0.41211 (15)	−0.0414 (3)	1.1175 (2)	0.0682 (8)	
H6A	0.3997	−0.0390	1.1906	0.102*	
H6B	0.4625	0.0183	1.1242	0.102*	
H6C	0.4219	−0.1445	1.0987	0.102*	
N1	0.11391 (11)	−0.0037 (2)	0.93458 (18)	0.0598 (6)	
H1A	0.1063	−0.0665	0.9853	0.072*	
H1B	0.0703	0.0334	0.8814	0.072*	
N2	0.20317 (11)	0.1349 (2)	0.85719 (15)	0.0470 (5)	
H2N	0.1585	0.1678	0.8037	0.056*	
N3	0.25965 (11)	−0.0223 (2)	1.02128 (16)	0.0480 (5)	

### Atomic displacement parameters (Å^2^)

**Table:**

	*U*^11^	*U*^22^	*U*^33^	*U*^12^	*U*^13^	*U*^23^
Cl	0.0469 (4)	0.0883 (5)	0.0535 (4)	0.0071 (3)	0.0131 (3)	0.0016 (3)
C1	0.0433 (12)	0.0489 (13)	0.0451 (14)	−0.0002 (11)	0.0144 (11)	−0.0052 (11)
C2	0.0516 (13)	0.0483 (13)	0.0459 (13)	−0.0037 (11)	0.0193 (11)	−0.0071 (11)
C3	0.0415 (12)	0.0603 (15)	0.0579 (16)	−0.0074 (11)	0.0190 (11)	−0.0044 (13)
C4	0.0447 (13)	0.0536 (14)	0.0469 (14)	0.0019 (11)	0.0117 (11)	−0.0066 (11)
C5	0.0717 (16)	0.0674 (16)	0.0581 (16)	−0.0057 (14)	0.0280 (13)	0.0069 (14)
C6	0.0487 (13)	0.0823 (19)	0.0663 (19)	0.0064 (14)	0.0073 (13)	0.0103 (15)
N1	0.0407 (11)	0.0734 (15)	0.0629 (15)	−0.0037 (10)	0.0129 (10)	0.0083 (10)
N2	0.0434 (10)	0.0530 (11)	0.0428 (11)	0.0034 (9)	0.0107 (8)	0.0017 (9)
N3	0.0425 (10)	0.0528 (12)	0.0467 (12)	0.0015 (9)	0.0110 (9)	0.0027 (9)

### Geometric parameters (Å, º)

**Table:**

C1—N1	1.325 (3)	C5—H5A	0.9600
C1—N3	1.331 (3)	C5—H5B	0.9600
C1—N2	1.356 (3)	C5—H5C	0.9600
C2—N2	1.356 (3)	C6—H6A	0.9600
C2—C3	1.359 (3)	C6—H6B	0.9600
C2—C5	1.486 (3)	C6—H6C	0.9600
C3—C4	1.395 (3)	N1—H1A	0.8600
C3—H3A	0.9300	N1—H1B	0.8600
C4—N3	1.333 (3)	N2—H2N	0.8600
C4—C6	1.494 (3)		
			
N1—C1—N3	119.3 (2)	H5A—C5—H5C	109.5
N1—C1—N2	118.9 (2)	H5B—C5—H5C	109.5
N3—C1—N2	121.8 (2)	C4—C6—H6A	109.5
N2—C2—C3	117.2 (2)	C4—C6—H6B	109.5
N2—C2—C5	117.3 (2)	H6A—C6—H6B	109.5
C3—C2—C5	125.4 (2)	C4—C6—H6C	109.5
C2—C3—C4	119.0 (2)	H6A—C6—H6C	109.5
C2—C3—H3A	120.5	H6B—C6—H6C	109.5
C4—C3—H3A	120.5	C1—N1—H1A	120.0
N3—C4—C3	122.7 (2)	C1—N1—H1B	120.0
N3—C4—C6	116.7 (2)	H1A—N1—H1B	120.0
C3—C4—C6	120.6 (2)	C2—N2—C1	121.99 (18)
C2—C5—H5A	109.5	C2—N2—H2N	119.0
C2—C5—H5B	109.5	C1—N2—H2N	119.0
H5A—C5—H5B	109.5	C1—N3—C4	117.2 (2)
C2—C5—H5C	109.5		

### Hydrogen-bond geometry (Å, º)

*Cg1* is the centroid of the C1–C4/N2/N3 ring.

**Table:**

*D*—H···*A*	*D*—H	H···*A*	*D*···*A*	*D*—H···*A*
N1—H1*A*···Cl^i^	0.86	2.42	3.260 (2)	167
N1—H1*B*···Cl^ii^	0.86	2.57	3.262 (2)	138
N2—H2*N*···Cl	0.86	2.22	3.042 (2)	161
C5—H5*A*···*Cg*1^iii^	0.96	3.00	3.446 (3)	110

Symmetry codes: (i) *x*, −*y*, *z*+1/2; (ii) −*x*, *y*, −*z*+3/2; (iii) −*x*+1/2, *y*+1/2, −*z*+3/2.
